# Microalbuminúria e seu Significado Prognóstico em Pacientes com Insuficiência Cardíaca Aguda com Fração de Ejeção Preservada, Intermediária e Reduzida

**DOI:** 10.36660/abc.20201144

**Published:** 2022-01-11

**Authors:** Ömer Doğan Alataş, Murat Biteker, Ahmet Demir, Birdal Yıldırım, Ethem Acar, Kemal Gökçek, Aysel Gökçek

**Affiliations:** 1 Muğla Sıtkı Koçman University Training and Research Hospital Department of Emergency Medicine Mugla Turquia Muğla Sıtkı Koçman University , Training and Research Hospital , Department of Emergency Medicine , Mugla – Turquia; 2 Muğla Sıtkı Koçman University Faculty of Medicine Department of Cardiology Mugla Turquia Muğla Sıtkı Koçman University , Faculty of Medicine , Department of Cardiology , Mugla – Turquia

**Keywords:** Albuminúria/fisiopatologia, Prognóstico, Insuficiência Cardíaca, Volume Sistólico, Hospitalização, Adultos, Mortalidade

## Abstract

**Fundamento:**

A prevalência e o significado da microalbuminúria não foram bem estudados em pacientes com diferentes subtipos de insuficiência cardíaca.

**Objetivo:**

A prevalência e o significado da microalbuminúria não foram bem estudados em pacientes com diferentes subtipos de insuficiência cardíaca. Portanto, nosso objetivo foi investigar a frequência e o valor prognóstico da microalbuminúria em pacientes hospitalizados por insuficiência cardíaca aguda (ICA) com fração de ejeção preservada (ICFEp), fração de ejeção de faixa média (ICFEfm) e fração de ejeção reduzida (ICFEr).

**Métodos:**

Todos os pacientes adultos consecutivos encaminhados ao hospital devido a ICA entre junho de 2016 e junho de 2019 foram inscritos. A microalbuminúria é definida como o nível de albumina urinária para relação de creatinina (AURC) na faixa de 30–300 mg/g. A mortalidade hospitalar foi o critério de valoração deste estudo.

**Resultados:**

Dos 426 pacientes com ICA (idade média de 70,64 ± 10,03 anos, 53,3% do sexo feminino), 50% tinham ICFEr, 38,3% tinham ICFEp e 11,7% tinham ICFEfm na apresentação. A prevalência de microalbuminúria foi de 35,2%, 28,8% e 28,0% em ICFEr, ICFEp e ICFEfm, respectivamente. Um total de 19 (4,5%) pacientes morreram durante o curso intra-hospitalar, e a mortalidade intra-hospitalar foi maior em pacientes com ICFEr (6,6%) em comparação com pacientes com ICFEr (2,5%) e ICFEfm (2,0%). A análise multivariada mostrou que a presença de microalbuminúria previu mortalidade intra-hospitalar em pacientes com ICFEr e ICFEfm, mas não em ICFEp.

**Conclusão:**

Embora a microalbuminúria fosse comum em todos os subgrupos de pacientes com ICA, descobriu-se que ela prediz o prognóstico apenas em pacientes com ICFEr e ICFEfm.

## Introdução

A insuficiência cardíaca (IC) foi classificada em três grupos com base na fração de ejeção do ventrículo esquerdo (FEVE) nas diretrizes atuais; IC com FE reduzida (ICFEr), IC com FE de faixa média (ICFEfm) e IC com FE preservada (ICFEp). ^1^ A insuficiência cardíaca aguda (ICA), que pode ser desenvolvida em todos os tipos de IC, é uma causa significativa de mortalidade e custos de saúde em países industrializados e em desenvolvimento. ^2 , 3^ Apesar dos avanços no manejo da ICA nas últimas décadas, 4% a 7% dos pacientes morrem durante a internação, e metade deles morre em cinco anos. ^4 , 5^ Portanto, a predição precoce da mortalidade é essencial para o manejo de pacientes com ICA, e existem muitas variáveis clínicas e laboratoriais que predizem a mortalidade na ICA. ^6 - 8^

Embora a disfunção renal também tenha sido associada ao aumento do risco de mortalidade na ICFEp , ^9^ estudos anteriores apresentaram achados conflitantes sobre a importância da doença renal crônica na ICFEp em comparação com a ICFEr; ^10^ e a importância das funções renais na ICFEfm não é clara. O aumento da excreção urinária de albumina, que pode ser um marcador de inflamação, disfunção endotelial e ativação do sistema renina-angiotensina, é um preditor de mortalidade e eventos adversos na população em geral, ^12^ em pacientes com diabetes ^13^ e hipertensão. ^14^ A relação albumina/creatinina urinária (AURC) em uma amostra de urina aleatória é aceita como um método mais útil para avaliar as funções renais e evita limitações de outros testes, como a taxa de filtração glomerular. ^15^ Na insuficiência cardíaca crônica, mesmo a disfunção renal leve, determinada pela presença de microalbuminúria (definida como níveis de albumina urinária maiores ou iguais a 30-300 mg na coleta de urina de 24 h ou AURC de> 30-300 mg/g em amostra aleatória de urina), está associada a resultados adversos. ^16^ Existem, no entanto, poucos relatórios que examinaram o efeito prognóstico do AURC em pacientes com ICA. Além disso, a prevalência e a significância da microalbuminúria não foram comparadas em ICFEr, ICFEfm e ICFEp. Portanto, nosso objetivo foi examinar a prevalência e a importância da microalbuminúria em pacientes com ICA secundária a ICFEr, ICFEfm e ICFEp.

## Métodos

Os dados de pacientes consecutivos hospitalizados por ED devido a ICA entre junho de 2016 e junho de 2019 foram registrados retrospectivamente. Este estudo foi conduzido no Hospital Universitário Muğla Sıtkı Koçman e aprovado pelo conselho de revisão institucional local.

### Critério de inclusão

Todos os pacientes adultos (≥ 18 anos) admitidos em nosso pronto-socorro com sinais e/ou sintomas de ICA e com níveis aumentados de peptídeo natriurético tipo B-N-Terminal (NT-proBNP) foram incluídos.

### Critério de exclusão

Pacientes que não tiveram avaliação de AURC, LVEF ou NT-proBNP na admissão, pacientes com idade <18 anos, pacientes em diálise e pacientes com alta o domicílio foram excluídos.

### Coleta de dados e definições

Os pacientes foram divididos em três grupos de acordo com a FEVE; pacientes com FEVE <50% foram definidos como ICFEr, pacientes com FEVE de 40-49% foram descritos como ICFEfm e pacientes com FEVE <40% foram definidos como ICFE. Além disso, os critérios ecocardiográficos de disfunção diastólica ou doença cardíaca estrutural também foram necessários para determinar a ICFEP.

As características demográficas e as comorbidades dos pacientes foram coletadas e anotadas no banco de dados do hospital. As definições das variáveis demográficas são fornecidas na Tabela 1 . Além disso, amostras de sangue e urina foram obtidas na admissão, incluindo NT-proBNP e níveis estimados da taxa de filtração glomerular (eTFG). ^17^

**Tabela 1:** – Dados demográficos e características dos pacientes

	ICFEr (n = 213)	ICFEfm (n = 50)	ICFEp (n = 163)	valor p
**Sexo feminino**	**110 (51,6)**	**22 (44,0)**	**95 (58,3)**	**<0,001**
**Idade**	**68,09 ± 9,58**	**70,85 ± 10,15**	**72,83 ± 10,70**	**0,015**
**Fumar**	**40 (18,8)**	**10 (20,0)**	**30 (18,4)**	**0,344**
**Uso de álcool**	**10 (4,7)**	**3 (6,0)**	**8 (4,9)**	**0,632**
**Índice de massa corporal, kg/m ^2^**	**27,56 ± 5,66**	**28,98 ± 5,92**	**29,43 ± 6,24**	**0,004**
**Comorbidades**				
Fibrilação atrial	65 (30,5)	15 (30,0)	50 (30,7)	0,845
Hipertensão	160 (75,2)	38 (76,0)	121 (74,2)	0,921
Diabetes mellitus	63 (29,6)	14 (28,0)	45 (27,6)	0,814
Doença renal crônica	25 (11,7)	5 (10,0)	16 (9,8)	0,623
Doença arterial coronária	95 (44,6)	26 (52,0)	66 (40,5)	0,014
Doença cerebrovascular	10 (4,7)	3 (6,0)	12 (7,4)	0,131
DPOC	21 (9,9)	5 (10,0)	15 (9,2)	0,755
**Sinais e sintomas**				
Dispneia, classe NYHA III/IV	171 (80,3)	42 (84,0)	134 (82,2)	0,510
Palpitação	130 (61,1)	30 (60,0)	105 (64,4)	0,212
Inchaço do tornozelo	70 (32,9)	15 (30,0)	51 (31,3)	0,815
Dor no peito	60 (28,2)	20 (40,0)	43 (26,4)	0,004
**Exame físico**				
Pressão arterial sistólica, mmHg	122,5 ± 15,41	131,22 ± 20,66	132,30 ± 20,11	0,001
Pressão arterial diastólica, mmHg	79,12 ± 11,96	80,10 ± 12,07	80,65 ± 11,86	0,109
Frequência cardíaca, bpm	88,75 ± 18,23	82,36 ± 18,05	82,55 ± 17,98	<0,001
Crepitações pulmonares	160 (75,2)	37 (74,0)	119 (73,0)	0,081
**Laboratório**				
NT-ProBNP, pg/ml	5859 (1896 - 11857)	3421 (1104-8455)	2544 (986 - 5487)	<0,001
Glicose, mg/dl	118 (94 - 158)	120 (96 - 161)	119 (95 - 159)	0,742
BUN, mg/dl	22 (18 - 37)	23 (17 - 35)	22 (16 - 36)	0,291
Creatinina sérica, mg/dl	1,2 (0,8 - 1,7)	1,2 (0,8 - 1,8)	1,1 (0,7 - 1,7)	0,366
Hemoglobina, g/dl	12,5 (10,1 - 14,5)	12,6 (10,5 - 13,5)	12,4 (10,8 - 14,2)	0,113
AURC	12,5 (5,9 - 1357,7)	10,3 (2,9 - 725,7)	10,1 (4,5 - 878,7)	0,001
eTFG (mL/min/1,73 m ^2^ )	68,7 ± 21,6	70,9 ± 21,3	70,7 ± 22,5	0,032
**Internação Hospitalar, mediana, dias**	**8**	**7**	**7**	**0,106**
**Mortalidade hospitalar**	**14 (6,6)**	**1 (2,0)**	**4 (2,5)**	**0,003**

*Os dados são apresentados como média ± desvio padrão, número (%) ou mediana e intervalo interquartil. ICFEr: insuficiência cardíaca com fração de ejeção reduzida; ICFEfm: insuficiência cardíaca com fração de ejeção de faixa média; ICFEp: insuficiência cardíaca com fração de ejeção preservada; NYHA: New York Heart Association; DPOC: doença pulmonar obstrutiva crônica; NT-proBNP: peptídeo natriurético do tipo pro B do terminal N; BUN: nitrogênio da ureia no sangue; AURC: relação albumina/creatinina urinária; eTFG: taxa de filtração glomerular estimada.*

A albuminúria foi definida de acordo com a relação albumina/creatinina na urina: normoalbuminúria: <30 mg/g, microalbuminúria: 30 -299 mg/ge macroalbuminúria:> 300 mg/g). O desfecho primário foi a mortalidade hospitalar.

### Análise estatística

Os dados foram analisados usando SPSS para Windows (versão 24; SPSS Inc, Chicago, IL). Um valor de P ≤0,05 foi considerado significativo. As análises de regressão univariada e multivariada foram realizadas para estudar o efeito de vários fatores de risco, incluindo microalbuminúria e macroalbuminúria, no desfecho primário.

## Resultados

**Figura 1:**
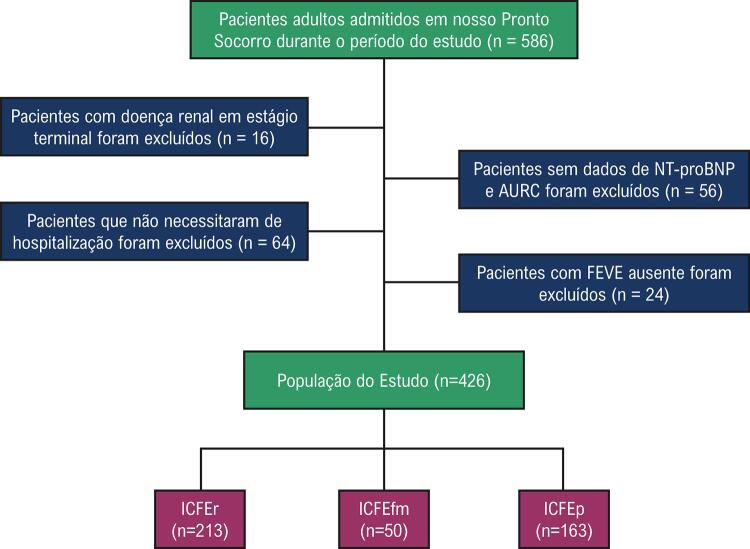
– Fluxograma do participante. NT-proBNP: peptídeo natriurético tipo B-N-Terminal; FEVE: fração de ejeção do ventrículo esquerdo; AURC: albumina urinária para relação de creatinina; ICFEr: insuficiência cardíaca com fração de ejeção reduzida; ICFEfm: insuficiência cardíaca com fração de ejeção de faixa média;ICFEp: insuficiência cardíaca com fração de ejeção preservada.

Um total de 586 pacientes adultos com ICA foram admitidos em nosso Pronto Socorro durante o período do estudo. No entanto, 24 pacientes sem dados de FEVE, 56 pacientes sem dados do NT-proBNP ou AURC, 64 pacientes que receberam alta para casa e 16 pacientes com doença renal em estágio terminal foram excluídos do estudo ( Figura 1 ). A população final do estudo incluiu 426 pacientes (idade média de 70,64 ± 10,03 anos, 53,3% do sexo feminino).

### Comparação das características basais em subgrupos de insuficiência cardíaca

Entre a população do estudo, 50% tinham ICFEr, 38,3% tinham ICFEp e 11,7% tinham ICFEfm.

As características basais dos pacientes são apresentadas na Table 1 . Os pacientes com ICFEP eram mais velhos, tinham um índice de massa corporal mais alto e eram mais propensos a serem mulheres. Os pacientes com ICFEr eram mais jovens, tinham níveis de NT-pro-BNP e AURC na admissão significativamente mais altos, tinham pressão arterial sistólica mais baixa, mas frequência cardíaca mais alta na apresentação. Pacientes com ICFEfm tinham um perfil de biomarcador intermediário e fenótipo intermediário para comorbidades. Pacientes com ICFEfm diferiam de ICFEp e ICFEr, pois eram mais frequentemente do sexo masculino e tinham maior probabilidade de ter história de doença arterial coronariana.

**Figura 2:**
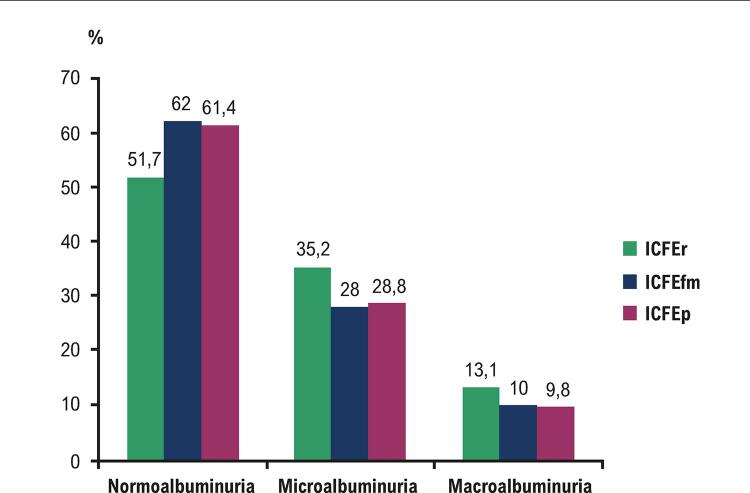
– Comparação da prevalência de normo-, micro- e macroalbuminúria em relação aos subtipos de insuficiência cardíaca. ICFEr: insuficiência cardíaca com fração de ejeção reduzida; ICFEfm: insuficiência cardíaca com fração de ejeção de faixa média;ICFEp: insuficiência cardíaca com fração de ejeção preservada.

Dos 426 pacientes, 185 (43,4%) tinham AURC aumentado na admissão; 136 pacientes tinham (31,9%) microalbuminúria, 49 pacientes tinham macroalbuminúria (11,5%) e 241 (56,6%) pacientes tinham normoalbuminúria. Não houve diferenças significativas na prevalência de normo-, micro- e macroalbuminúria em pacientes com ICFEp e ICFEr. No entanto, em comparação com ICFEp e ICFEfm, os pacientes com ICFEr eram mais propensos a ter micro e macroalbuminúria e eram menos propensos a normoalbuminúria ( Figura 2 ). A prevalência de microalbuminúria foi de 35,2%, 28,8% e 28,0% em ICFEr, ICFEp e ICFEfm, respectivamente. A prevalência de microalbuminúria foi de 13,1%, 9,8% e 10% em ICFEr, ICFEp e ICFEfm, respectivamente.

### Comparação de resultados

Não houve diferença no tempo de internação hospitalar entre os pacientes com ICFEp, ICFEfm ou ICFEr. Um total de 19 (4,5%) pacientes morreram durante o curso intra-hospitalar, e a mortalidade intra-hospitalar foi maior em pacientes com ICFEr (6,6%) em comparação para pacientes com ICFEp (2,5%) e ICFEfm (2,0%) (p = 0,004).

### Preditores de mortalidade hospitalar

A análise multivariada mostrou que o NT-pro-BNP e a macroalbuminúria foram associados à mortalidade hospitalar em todos os grupos com FEVE ( Table 2 ). Doença arterial coronariana, sexo masculino e diabetes mellitus predisseram mortalidade intra-hospitalar apenas em pacientes com ICFEfm, enquanto a fibrilação atrial previu mortalidade intra-hospitalar apenas em pacientes com ICFEr. A idade avançada foi um preditor independente de mortalidade hospitalar em pacientes com ICFEr e ICFEp.

**Tabela 2:** – Preditores de mortalidade hospitalar em subtipos de IC

	ICFEr		ICFEfm		ICFEp	
	OR (IC 95%)	Valor p	OR (IC 95%)	Valor p	OR (IC 95%)	Valor p
Doença arterial coronária	2,10 (1,55-3,04)	0,065	3,45 (1,23-5,67)	0,043	1,49 (1,14-5,34)	0,089
NT-ProBNP	2,68 (1,23-7,75)	<0,001	2,12 (1,34-3,45)	0,011	2,01 (0,09-3,23)	0,022
Idade (por 10 anos)	1,75 (1,13-3,45)	0,016	1,13 (0,80-1,51)	0,076	3,12 (1,38-4,81)	0,019
Diabetes mellitus	1,21 (0,81-1,43)	0,121	2,34 (1,03-4,16)	0,043	1,20 (0,89-2,55)	0,291
Microalbuminúria	1,94 (0,91-4,21)	<0,001	1,56 (1,19-3,45)	0,001	1,25 (1,12-1,68)	0,124
Macroalbuminúria	2,45 (1,34-5,65)	<0,001	1,92 (1,23-2,98)	0,024	1,66 (1,34-3,84)	0,032
Doença renal crônica	1,15 (1,01-1,33)	0,293	1,32 (1,11-2,77)	0,101	1,23 (0,82-1,56)	0,451
Fibrilação atrial	1,07 (0,83-1,42)	0,013	1,23 (0,89-1,55)	0,234	1,33 (1,18-2,01)	0,098
Sexo masculino	1,22 (0,83-1,88)	0,462	3,31 (1,13-4,23)	0,001	0,89 (0,66-1,39)	0,453

*ICFEr: insuficiência cardíaca com fração de ejeção reduzida; ICFEfm: insuficiência cardíaca com fração de ejeção de faixa média; ICFEp: insuficiência cardíaca com fração de ejeção preservada; NT-proBNP: peptídeo natriurético do tipo pro B do terminal N.*

### Microalbuminúria e prognóstico

A presença de microalbuminúria na admissão foi associada à mortalidade intra-hospitalar em pacientes com ICFEr e ICFEfm, mas não em pacientes com ICFEp. Pacientes com microalbuminúria e macroalbuminúria tiveram risco 1,94 e 2,45 vezes maior, respectivamente, de mortalidade hospitalar em comparação com pacientes com normoalbuminúria na ICFEr. Em comparação com pacientes com normoalbuminúria, pacientes com microalbuminúria e macroalbuminúria tiveram 1,56 e 1,92 vezes maior risco de mortalidade hospitalar em ICFEfm, respectivamente.

## Discussão

Nosso estudo tem várias importantes implicações clínicas: (i) Dos pacientes hospitalizados com ICA, 50% tinham ICFEr, 11,7% tinham ICFEfm e 38,3% tinham ICFEp. (ii) 43,4% dos pacientes apresentavam AURC anormal na admissão ao PS. (iii) Os valores de NT-proBNP e AURC e as taxas de mortalidade hospitalar foram os mais elevados em pacientes com ICFEr. (iv) A prevalência de micro e macroalbuminúria em ICFEp foi semelhante a ICFEfm e menor do que ICFEr. (v) A prevalência de microalbuminúria foi de 35,2%, 28,8% e 28,0% em ICFEr, ICFEp e ICFEfm, respectivamente. (vi) A microalbuminúria previu mortalidade intra-hospitalar em ICFEfm e ICFEr, mas não em ICFEp.

As doenças cardiovasculares e renais compartilham comorbidades e fatores de risco semelhantes. Estudos de coorte extensos mostraram que o aumento do AURC está associado ao desenvolvimento de IC na população em geral. ^18 - 20^ No entanto, a maioria dos estudos descreveu a importância do AURC em ICFEr, e os estudos que examinam os subtipos de HF separadamente apresentam achados divergentes. ^21 , 22^ Em um estudo comunitário, Nayor et al., ^21^ descobriram que a microalbuminúria foi associada a um risco aumentado de ICFEr incidente, mas não ICFEp. ^21^ Em contraste, o estudo de coorte PREVEND mostrou que AURC mais alta estava mais fortemente associada a ICFEp incidente do que ICFEr. ^22^ Em uma pesquisa recente com 24433 pacientes, a associação entre AURC e ICFEp foi maior do que ICFEr após 9,3 anos de acompanhamento. ^23^

Os testes de função renal também estão associados a resultados adversos, independentemente da gravidade da doença em pacientes com IC estabelecida. No entanto, estudos que investigam o impacto da disfunção renal no prognóstico nos diferentes grupos de FEVE também apresentam resultados conflitantes. ^24 , 25^ Em uma metanálise, Damman et al., ^24^ mostraram que a disfunção renal crônica foi um preditor mais forte de mortalidade na ICFEp do que na ICFEr. ^24^ Em contraste, em uma metanálise de vinte e cinco estudos prospectivos, a disfunção renal foi um preditor mais forte de mortalidade em pacientes com ICFEr do que em ICFEp. ^25^ Ambas as metanálises definiram a doença renal crônica como um eTFG inferior a 60 ml/min /1,73m ^2^ , e os estudos que examinam o valor prognóstico da microalbuminúria ou AURC em pacientes com IC crônica com diferentes grupos de FEVE são muito mais limitados. ^26 - 30^ Em um estudo transversal, 72 pacientes com IC crônica foram inscritos, e a microalbuminúria foi observada em 40% dos pacientes com ICFEp e em 24% dos pacientes com ICFEr (p = 0,04). ^26^ No entanto, o impacto prognóstico da microalbuminúria não foi avaliado neste estudo. No estudo CHARM, que incluiu pacientes com IC crônica, a prevalência de micro e macroalbuminúria foi de 30% e 11%, respectivamente. ^27^ Ao estratificar em diferentes grupos de FEVE, 31% dos pacientes com FEVE ≤40% tinham microalbuminúria, e 10% tinham macroalbuminúria. Dos pacientes com FEVE> 40%, 29% tinham microalbuminúria e 12% macroalbuminúria. Os resultados do estudo CHARM também revelaram que a albuminúria foi um preditor de mortalidade. O risco associado ao AURC foi semelhante em pacientes com FEVE baixa e preservada. ^27^ No estudo GISSI-HF, micro e macroalbuminúria foram observados em 19,9% e 5,4% dos pacientes, respectivamente. O AURC previu mortalidade independentemente em pacientes com IC crônica. ^28^ No entanto, como 90,8% dos pacientes com GISSI-HF tinham FEVE ≤ 40%, uma análise separada para diferentes grupos de FEVE não foi realizada. No estudo CHART-2, 2.039 pacientes com IC crônica foram inscritos. ^29^ Os autores mostraram que não apenas a microalbuminúria, mas também a microalbuminúria subclínica, que foi definida como AURC 10,2–27,3 mg/g, estava significativamente associada a eventos cardiovasculares adversos em comparação com a normoalbuminúria, particularmente em pacientes com eTFG preservada ou levemente reduzido. ^28^ O estudo TOPCAT incluiu apenas pacientes com ICFEp para investigar o benefício da terapia com espironolactona. ^30^ Em uma análise de subgrupo do estudo TOPCAT, micro e macroalbuminúria conferiram um risco aumentado de 1,47 e 1,67 vezes para desfechos primários em ICFEp. ^30^

Embora se espere que a prevalência de disfunção renal seja maior em pacientes com ICA do que em pacientes com IC crônica, poucos estudos avaliaram a albuminúria no contexto de ICA. Em um estudo prospectivo de 115 pacientes com ICA, Koyama et al., ^31^ mostraram que 69% dos pacientes tinham AURC anormal na admissão (27% tinham macroalbuminúria, 42% tinham microalbuminúria). ^31^ No entanto, no dia 7, 10% dos pacientes tinham macroalbuminúria e 30% tinham microalbuminúria. A resolução do AURC foi associada a diminuições nos níveis de NT-proBNP. ^31^ A frequência de AURC anormal na admissão foi de 43,4% em nosso estudo, menor do que o estudo de Koyama e colegas. Essa diferença pode ser devido à idade mais jovem e menor carga de comorbidades em nosso estudo.

Nosso estudo demonstrou que a microalbuminúria na admissão no Pronto Socorro é um preditor independente de mortalidade intra-hospitalar em ICFEfm e ICFEr, mas não em ICFEp. Em ICFEp, o prognóstico pode estar mais relacionado a comorbidades do que em ICFEfm e ICFEr, onde com subsequente disfunção renal pode ser mais pronunciada. A relação entre IC e albuminúria é complexa. É de natureza bidirecional, e os mecanismos responsáveis pela relação da microalbuminúria e o prognóstico na ICFEr e ICFEfm merecem investigações adicionais.

### Limitações do estudo

Nosso estudo é limitado por seu desenho retrospectivo e por ter sido realizado em um único centro. Como as mudanças diárias no AURC não foram registradas, não pudemos examinar a relação entre as alterações no AURC e o prognóstico. Uma amostra única de urina foi usada para determinar o AURC, que pode flutuar.

## Conclusões

Em pacientes com ICA, a microalbuminúria na admissão está associada ao aumento da mortalidade intra-hospitalar em ICFEfm e ICFEr. Mais estudos prospectivos são necessários para explorar o papel do AURC como um marcador prognóstico na ICA.

## References

[1] Ponikowski P, Voors AA, Anker SD, Bueno H, Cleland JGF, Coats AJS, et al. 2016 ESC Guidelines for the diagnosis and treatment of acute and chronic heart failure: The Task Force for the diagnosis and treatment of acute and chronic heart failure of the European Society of Cardiology (ESC). Developed with the special contribution of the Heart Failure Association (HFA) of the ESC. Eur J Heart Fail. 2016;18(27):891-975. 10.1002/ejhf.592 27207191

[2] Sasaki N, Kunisawa S, Ikai H, Imanaka Y, Differences between determinants of in-hospital mortality and hospitalisation costs for patients with acute heart failure: a nationwide observational study from Japan. BMJ Open. 2017;7(3):e013753. 10.1136/bmjopen-2016-013753 PMC5372154 28336741

[3] Salam A, Sulaiman K, Alsheikh A, Singh R, AlHabib KF, Al-zakwani KF, et al. Precipitating Factors for Hospitalization with Heart Failure: Prevalence and Clinical Impact Observations from the Gulf CARE (Gulf aCute heArt failuRe rEgistry).Med Princ Pract. 2019;2020;29(3):270-8. doi: 10.1159/000503334. 10.1159/000503334 PMC7315136 31522185

[4] Adams Jr KF, Fonarow GC, Emerman CL, Lejemtel TH, Costanzo MR, Abraham WT, et al. ADHERE Scientific Advisory Committee and Investigators. Characteristics and outcomes of patients hospitalized for heart failure in the United States: rationale, design, and preliminary observations from the first 100,000 cases in the Acute Decompensated Heart Failure National Registry (ADHERE). Am Heart J.2005;149(2):209–16. 10.1016/j.ahj.2004.08.005 15846257

[5] Fonarow GC, Abraham WT, Albert N, Gattis W, Gheorghiade M, Greenberg B, et al. Impact of evidence-based heart failure therapy use at hospital discharge on treatment rates during follow-up: a report from the Organized Program to Initiate Lifesaving Treatment in Hospitalized Patients With Heart Failure(OPTIMIZE-HF). J Am Coll Cardiol. 2005;45:345A.

[6] Moleerergpoom W, Hengrussamee K, Piyayotai D, Jintapakorn W, Sukhum P, Kunjara-Na-Ayudhya R, et al. Predictors of in-hospital mortality in acute decompensated heart failure (Thai ADHERE). J Med Assoc Thai. 2013;96(2):157-64. 23936980

[7] Parissis JT, Mantziari L, Kaldoglou N, Ikonomidis I, Nikolaou M, Mebazaa N, et al. Gender-related differences in patients with acute heart failure: management and predictors of in-hospital mortality. Int J Cardiol. 2013;168(1):185-9. 10.1016/j.ijcard.2012.09.096 23041090

[8] Castello LM, Molinari L, Renghi A, Peruzzi E, Capponi A, Avanzi GC, et al.Acute decompensated heart failure in the emergency department: Identification of early predictors of outcome. Medicine (Baltimore). 2017;96(27):e7401. 10.1097/MD.0000000000007401 PMC5502168 28682895

[9] Givertz MM, Postmus D, Hillege HL, Mansoor GA, Massie BM, Davison BA, et al. Renal function trajectories and clinical outcomes in acute heart failure.Circ Heart Fail. 2014;7(5):59-67. 10.1161/CIRCHEARTFAILURE.113.000556 24281137

[10] Casado J, Sánchez M, Garcés V, Manzano L, Cerqueiro JM, Epelde F, et al; RICA Investigators Group. Influence of renal dysfunction phenotype on mortality in decompensated heart failure with preserved and mid-range ejection fraction. Int J Cardiol. 2017;243:332-9. 10.1016/j.ijcard.2017.05.048 28528982

[11] Park CS, Park JJ, Oh IY, Yoon CH, Choi DJ, Park HA, et al.; KorHF Investigators.Relation of Renal Function with Left Ventricular Systolic Function and NT-proBNP Level and Its Prognostic Implication in Heart Failure with Preserved versus Reduced Ejection Fraction: an analysis from the Korean Heart Failure (KorHF) Registry. Korean Circ J. 2017;47(5):727-41. 10.4070/kcj.2017.0050 PMC5614949 28955391

[12] Chong J, Fotheringham J, Tomson C, Ellam T. Renal albumin excretion in healthy young adults and its association with mortality risk in the US population. Nephrol Dial Transplant. 2020;35(3):458-64. doi: 10.1093/ndt/gfy242. 10.1093/ndt/gfy242 30085245

[13] Lunetta M, Infantone L, Calogero AE, Infantone E. Increased urinary albumin excretion is a marker of risk for retinopathy and coronary heart disease in patients with type 2 diabetes mellitus. Diabetes Res Clin Pract. 1998;40(1):45-51. 10.1016/s0168-8227(98)00024-2 9699090

[14] Wachtell K, Ibsen H, Olsen MH, Borch-Johnsen K, Lindholm LH, Mogensen CE, et al. Albuminuria and cardiovascular risk in hypertensive patients with left ventricular hypertrophy: the LIFE study. Ann Intern Med.2003;139(92): 901–6. 10.7326/0003-4819-139-11-200312020-00008 14644892

[15] Takahashi S, Tanaka F, Yonekura Y, Tanno K, Ohsawa M, Sakata K, et al. The urine albumin-creatinine ratio is a predictor for incident long-term care in a general population. PLoS One. 2018;13(3):e0195013. 10.1371/journal.pone.0195013 PMC5874057 29590199

[16] Villacorta H, Ferradaes P de V, Mesquita ET, Nobrega AC. Microalbuminuria is an independent prognostic marker in patients with chronic heart failure. Arq Bras Cardiol. 2012;98(1):62-9. doi:10.1590/s0066-782x2011005000120. 10.1590/s0066-782x2011005000120 22328318

[17] Levey AS, Coresh J, Greene T, Marsh J, Stevens LA, Kusek JW, et al. Chronic Kidney Disease Epidemiology Collaboration. Expressing the Modification of Diet in Renal Disease Study equation for estimating glomerular filtration rate with standardized serum creatinine values. Clin Chem. 2007;53(4):766–72. 10.1373/clinchem.2006.077180 17332152

[18] Arnlov J, Evans JC, Meigs JB, Wang T, Fox C, Levy D, et al. Low-grade albuminuria and incidence of cardiovascular disease events in nonhypertensive and nondiabetic individuals: the Framingham Heart Study. Circulation. 2005; 112(7):969–75. 10.1161/CIRCULATIONAHA.105.538132 16087792

[19] Kistorp C, Raymond I, Pedersen F, Gustafsson F, Faber J, Hildebrandt P. N-terminal pro-brain natriuretic peptide, C-reactive protein, and urinary albumin levels as predictors of mortality and cardiovascular events in older adults. JAMA. 2005;293(13):1609–16. 10.1001/jama.293.13.1609 15811980

[20] Ingelsson E, Sundstrom J, Lind L, Riserus U, Larsson A, Basu S, et al. Low-grade albuminuria and the incidence of heart failure in a community-based cohort of elderly men. Eur Heart J. 2007; 28(14):1739–45. 10.1093/eurheartj/ehm130 17495987

[21] Nayor M, Larson MG, Wang N, Santhanakrishnan R, Lee DS, Tsao CW, et al.The association of chronic kidney disease and microalbuminuria with heart failure with preserved vs. reduced ejection fraction. Eur J Heart Fail. 2017;19(5):615-23. 10.1002/ejhf.778 PMC5423843 28217978

[22] Brouwers FP, de Boer RA, van der Harst P, Voors A, Gansevoort RT, Bakker SJ, et al. Incidence and epidemiology of new onset heart failure with preserved vs. reduced ejection fraction in a community-based cohort: 11-year follow-up of PREVEND. Eur Heart J. 2013;34(19):424-31. 10.1093/eurheartj/eht066 23470495

[23] Bailey LN, Levitan EB, Judd SE, Sterling MR, Goyal P, Cushman M, et al. Association of Urine Albumin Excretion With Incident Heart Failure Hospitalization in Community-Dwelling Adults. JACC Heart Fail. 2019;7(5):394-401. 10.1016/j.jchf.2019.01.016 PMC6544368 31047019

[24] Damman K, Valente MA, Voors AA, O’Connoor CM, van Veldhuisen DJ, Hillege HL. Renal impairment, worsening renal function, and outcome in patients with heart failure: an updated meta-analysis. Eur Heart J. 2014;35(7):455-69. 10.1093/eurheartj/eht386 24164864

[25] McAlister FA, Ezekowitz J, Tarantini L, Squire I, Komajda M, Bayes-Genis A, et al. Renal dysfunction in patients with heart failure with preserved versus reduced ejection fraction: impact of the new Chronic Kidney Disease-Epidemiology Collaboration Group formula. Circ Heart Fail. 2012;5(3):309-14. 10.1161/CIRCHEARTFAILURE.111.966242 22441773

[26] Orea-Tejeda A, Colín-Ramírez E, Hernández-Gilsoul T, Castillo-Martinez L, Abasta-Jimemnez M, Asensio Lafuente E, et al. Microalbuminuria in systolic and diastolic chronic heart failure patients.Cardiol J. 2008;15(2):143-9. 18651398

[27] Jackson CE, Solomon SD, Gerstein HC, Zetterstrand S, Olofsson B, Michelson EL, et al. Albuminuria in chronic heart failure: prevalence and prognostic importance.Lancet. 2009;374(9689):543-50. 10.1016/S0140-6736(09)61378-7 19683640

[28] Masson S, Latini R, Milani V, Moretti L, Rossi MG, Carbonieri E, et al. GISSI-HF Investigators. Prevalence and prognostic value of elevated urinary albumin excretion in patients with chronic heart failure: data from the GISSI-Heart Failure trial. Circ Heart Fail. 2010;3(1):65-72. 10.1161/CIRCHEARTFAILURE.109.881805 19850697

[29] Miura M, Sakata Y, Miyata S, Nochioka K, Takada T, Tadaki S, et al. CHART-2 Investigators. Prognostic impact of subclinical microalbuminuria in patients with chronic heart failure.Circ J.2014;78(9):2890-8. 25421233

[30] Selvaraj S, Claggett B, Shah SJ, E,Anand I, Rouleau JL, O’Meara E, et al.Prognostic Value of Albuminuria and Influence of Spironolactone in Heart Failure With Preserved Ejection Fraction. Circ Heart Fail. 2018;11(11):e00528. 10.1161/CIRCHEARTFAILURE.118.005288 PMC6594383 30571191

[31] Koyama S, Sato Y, Tanada Y, Fujiwara H, Takatsu Y.Early evolution and correlates of urine albumin excretion in patients presenting with acutely decompensated heart failure. Circ Heart Fail. 2013;6(2):227-32. 10.1161/CIRCHEARTFAILURE.112.000152 23395932

