# Supplementary material for: Microalbuminúria e seu Significado Prognóstico em Pacientes com Insuficiência Cardíaca Aguda com Fração de Ejeção Preservada, Intermediária e Reduzida
Source: Arq Bras Cardiol. 2022 Jan 11;118(4):703–9. [Article in Portuguese] doi: 10.36660/abc.20201144 (PMC9007018; doi:10.36660/abc.20201144)
Supplement: Translation

# Translation: Microalbuminuria and its Prognostic Significance in Patients with Acute Heart Failure with Preserved, Mid-Range, and Reduced Ejection Fraction

**Authors:** Ömer Doğan Alataş, Murat Biteker, Ahmet Demir, Birdal Yıldırım, Ethem Acar, Kemal Gökçek, Aysel Gökçek

**Affiliations:** 1 Turkey Muğla Sıtkı Koçman University, Training and Research Hospital, Department of Emergency Medicine, Mugla – Turkey; 2 Turkey Muğla Sıtkı Koçman University, Faculty of Medicine, Department of Cardiology, Mugla – Turkey

**Keywords:** Albuminuria/physiopathology, Prognosis, Heart Failure, Stroke Volume, Hospitalization, Adults, Mortality

## Abstract

**Background:**

The prevalence and significance of microalbuminuria have not been well studied in patients with different heart failure subtypes.

**Objective:**

The prevalence and significance of microalbuminuria have not been well studied in patients with different heart failure subtypes. Therefore, we aimed to investigate the frequency and prognostic value of microalbuminuria in patients hospitalized for acute heart failure (AHF) with preserved ejection fraction (HFpEF), mid-range ejection fraction (HFmrEF), and reduced ejection fraction (HFrEF).

**Methods:**

All consecutive adult patients referred to the hospital due to AHF between June 2016 and June 2019 were enrolled. Microalbuminuria is defined as urinary albumin to creatinine ratio (UACR) level in the range of 30–300 mg/g. Hospital mortality was the endpoint of this study

**Results:**

Of the 426 AHF patients (mean age 70.64 ± 10.03 years, 53.3 % female), 50% had HFrEF, 38.3% had HFpEF, and 11.7% had HFmrEF at presentation.The prevalence of microalbuminuria was 35.2%, 28.8%, and 28.0% in HFrEF, HFpEF, and HFmrEF, respectively. A total of 19 (4.5%) patients died during the in-hospital course, and in-hospital mortality was higher in HFrEF patients (6.6%) compared to patients with HFpEF (2.5%) and HFmrEF (2.0%). Multivariate analysis showed that the presence of microalbuminuria predicted in-hospital mortality in patients with HFrEF and HFmrEF but not in HFpEF.

**Conclusion:**

Although microalbuminuria was common in all subgroups of AHF patients, it has been found to predict prognosis only in patients with HFrEF and HFmrEF.

## Introductıon

Heart failure (HF) has been classified into three groups based on left ventricular ejection fraction (LVEF) in current guidelines; HF with reduced EF (HFrEF), HF with mid-range EF (HFmrEF), and HFwith preserved EF (HFpEF).^1^Acute heart failure (AHF), which can be developed in all types of HF, is a significant cause of mortality and healthcare costs in industrialized and developing countries.^2 , 3^Despite the advances in the management of AHF in the last decades, 4% to 7% of the patients die during hospitalization, and half of them die within five years.^4 , 5^Therefore, early prediction of mortality is essential for the management of patients with AHF, and there are many clinical and laboratory variables that predict mortality in AHF.^6 - 8^

Although kidney dysfunction has also been associated with increased mortality risk in AHF,^9^previous studies had conflicting findings regarding the importance of chronic renal disease in HFpEF compared to HfrEF,^10 , 11^and the significance of renal functions in HFmrEF is unclear. Increased urinary albumin excretion, which might be a marker of inflammation, endothelial dysfunction, and activated the renin-angiotensin system, is a predictor of mortality and adverse events in the general population,^12^in patients with diabetes,^13^and hypertension.^14^The urinary albumin/creatinine ratio (UACR) in a random urine specimen is accepted as a more helpful method for evaluating renal functions. It avoids limitations of other tests such as glomerular filtration rate.^15^In chronic heart failure, even mild renal dysfunction, determined by the presence of microalbuminuria (defined as urinary albumin levels of more than or equal to 30-300 mg in 24 h urine collection or UACR of >30-300 mg/g in random spot urine sample), is an associated with adverse outcomes.^16^There are, however, few reports that examined the prognostic effect of UACRin patients with AHF. Furthermore, the prevalence and significance of microalbuminuria have not been compared in HFrEF, HFmrEF, and HFpEF. Therefore, we aimed to examine the prevalence and importance of microalbuminuria in patients with AHF secondary to HFrEF, HFmrEF, and HFpEF.

## Methods

Data of consecutive patients hospitalized through EDdue to AHF between June 2016 and June 2019 were retrospectively recorded. This study was conducted in Muğla Sıtkı Koçman University Hospital, and approved by the local institutional review board.

### Inclusion Criteria

All adult patients (≥ 18 years)admitted to our ED with signs and/or symptoms of AHF and with increased N-Terminal pro-B-Type Natriuretic Peptide (NT-proBNP) levels were included.

### Exclusion Criteria

Patients who did not have UACR, LVEF, or NT-proBNP evaluation at admission, patients aged <18 years, dialysis patients, and patients discharged to home were excluded.

### Data Collection and Definitions

Patients were divided into three groups according to LVEF; patients with an LVEF <50% were defined as HFrEF, patients with an LVEF 40-49% were described as HFmrEF and patients with an LVEF < 40% were defined as HFpEF. In addition, echocardiographic criteria of diastolic dysfunction or structural heart disease were also required to determine HFpEF.

Patients’demographic characteristics and comorbidities were collected and noted from the hospital database. Definitions of demographic variables are given in the Table 1 . In addition, blood and urine samples were obtained at admission, including NT-proBNP and estimated glomerular filtration rate (eGFR) levels.^17^

**Table 1:** – Patient demographics and characteristics

	HFrEF (n=213)	HFmrEF (n=50)	HFpEF (n=163)	p value
**Female sex**	**110 (51.6)**	**22 (44.0)**	**95 (58.3)**	**<0.001**
**Age, years**	**68.09 ± 9.58**	**70.85 ± 10.15**	**72.83 ± 10.70**	**0.015**
**Smoking**	**40 (18.8)**	**10 (20.0)**	**30 (18.4)**	**0.344**
**Alcohol use**	**10 (4.7)**	**3 (6.0)**	**8 (4.9)**	**0.632**
**Body mass index, kg/m^2^**	**27.56 ± 5.66**	**28.98 ± 5.92**	**29.43 ± 6.24**	**0.004**
**Comorbidities**				
Atrial fibrillation	65 (30.5)	15 (30.0)	50 (30.7)	0.845
Hypertension	160 (75.2)	38 (76.0)	121 (74.2)	0.921
Diabetes mellitus	63 (29.6)	14 (28.0)	45 (27.6)	0.814
Chronic renal disease	25 (11.7)	5 (10.0)	16 (9.8)	0.623
Coronary artery disease	95 (44.6)	26 (52.0)	66 (40.5)	0.014
Cerebrovascular disease	10 (4.7)	3 (6.0)	12 (7.4)	0.131
COPD	21 (9.9)	5 (10.0)	15 (9.2)	0.755
**Signs and Symptoms**				
Dyspnea, NYHA class III/IV	171 (80.3)	42 (84.0)	134 (82.2)	0.510
Palpitation	130 (61.1)	30 (60.0)	105 (64.4)	0.212
Ankle swelling	70 (32.9)	15 (30.0)	51 (31.3)	0.815
Chest pain	60 (28.2)	20 (40.0)	43 (26.4)	0.004
**Physical Exam**				
Systolic blood pressure, mmHg	122.5 ± 15.41	131.22 ± 20.66	132.30 ± 20.11	0.001
Diastolic blood pressure, mmHg	79.12 ± 11.96	80.10 ± 12.07	80.65 ± 11.86	0.109
Heart rate, bpm	88.75 ± 18.23	82.36 ± 18.05	82.55± 17.98	<0.001
Pulmonary crepitations	160 (75.2)	37 (74.0)	119 (73.0)	0.081
**Laboratory**				
NT-proBNP, pg/ml	5859 (1896 - 11857)	3421 (1104 - 8455)	2544 (986 - 5487)	<0.001
Glucose, mg/dl	118 (94 - 158)	120 (96 - 161)	119 (95 - 159)	0.742
BUN, mg/dl	22 (18 - 37)	23 (17 - 35)	22 (16 - 36)	0.291
Serum creatinine, mg/dl	1.2 (0.8 - 1.7)	1.2 (0.8 - 1.8)	1.1 (0.7 - 1.7)	0.366
Hemoglobin, g/dl	12.5 (10.1 - 14.5)	12.6 (10.5 - 13.5)	12.4 (10.8 - 14.2)	0.113
UACR	12.5 (5.9 - 1357.7)	10.3 (2.9 - 725.7)	10.1 (4.5 - 878.7)	0.001
eGFR (mL/min/1.73 m^2^)	68.7 ± 21.6	70.9 ± 21.3	70.7 ± 22.5	0.032
**Hospitalstay,median,days**	**8**	**7**	**7**	0.106
**In-hospital mortality**	**14 (6.6)**	**1 (2.0)**	**4 (2.5)**	0.003

*Data are presented as mean ± standard deviation, number (%), or median and interquartile range. HfrEF: heart failure with reduced ejection fraction; HfmrEF: heart failure with mid-range ejection fraction; HfpEF: heart failure with preserved ejection fraction; NYHA: New York Heart Association; COPD: chronic obstructive pulmonary disease; NT-proBNP: N-terminal pro B-type natriuretic peptide; BUN: blood urea nitrogen; UACR: urinary albumin/creatinine ratio; eGFR: estimated glomerular filtration rate.*

The albuminuria was defined according to the urine albumin to creatinine ratio: normoalbuminuria: <30 mg/g, microalbuminuria: 30 -299 mg/g, and macroalbuminuria: > 300 mg/g).The primary end point was in-hospital mortality.

### Statistical analysis

Data were analyzed using SPSS for Windows (version 24; SPSS Inc, Chicago, IL). A p-value of ≤0.05 was considered significant. The univariate and multivariate regression analyses were performed to study the effect of various risk factors, including microalbuminuria and macroalbuminuria, on the primary outcome.

## Results

A total of 586 adult AHF patients were admitted to our ED during the study period. However, 24 patients without LVEF data, 56 patients without NT-proBNPor UACR data, 64 patients who were discharged to home, and 16 patients with end-stage renal disease were excluded from the study ( Figure 1 ). The final study population included 426 patients (mean age 70.64 ± 10.03 years, 53.3 % female).

**Figure 1:** – Participant flow chart. NT-proBNP: N-Terminal proB-Type Natriuretic Peptide; UACR: urinary albumin/creatinine ratio; LVEF: left ventricular ejection fraction; HfrEF: heart failure with reduced ejection fraction; HfmrEF: heart failure with mid-range ejection fraction; HfpEF: heart failure with preserved ejection fraction

### Comparison of baseline characteristics in heart failure subgroups

Among the study population, 50% had HFrEF, 38.3% had HFpEF, and 11.7% had HFmrEF.

The baseline characteristics of the patients are shown in Table 1 . Patients with HFpEF were older, had a higher body mass index, and were more likely to be female. Patients with HFrEF were younger, had significantly higher admission NT-pro-BNP and UACR levels, had lower systolic blood pressures but higher heart rates at presentation. Patients with HFmrEF had an intermediate biomarker profile and intermediate phenotype for comorbid diseases. Patients with HFmrEF differed from HFpEF and HFrEF, as they were more often male and more likely to have a history of coronary artery disease.

Of the 426 patients, 185 (43.4%) had increased UACR at admission; 136 patients had (31.9%) microalbuminuria, 49 patients had macroalbuminuria (11.5%), and 241 (56.6%) patients had normoalbuminuria. There were no significant differences in the prevalence of normo-, micro- and macroalbuminuria in patients with HFpEF and HFrEF. However, compared with HFpEF and HFmrEF, HFrEF patients were more likely to have micro- and macroalbuminuria and were less likely to normoalbuminuria ( Figure 2 ). The prevalence of microalbuminuria was 35.2%, 28.8%, and 28.0% in HFrEF, HFpEF, and HFmrEF, respectively. The prevalence of microalbuminuria was 13.1%, 9.8%, and 10% in HFrEF, HFpEF, and HFmrEF, respectively.

**Figure 2:** – Comparison of the prevalence of normo-, micro- and macroalbuminuria in relation to heart failure subtypes. HfrEF: heart failure with reduced ejection fraction; HfmrEF: heart failure with mid-range ejection fraction; HfpEF: heart failure with preserved ejection fraction

### Comparison of Outcomes

There was no difference in length of hospital stay between patients with HFpEF, HFmrEF or HFrEF.A total of 19 (4.5%) patients died during the in-hospital course, and in-hospital mortality was higher in HFrEF patients (6.6%) compared to patients with HFpEF (2.5%), and HFmrEF (2.0%) (p = 0.004).

### Predictors of In-hospital Mortality

Multivariate analysis showed that NT-pro-BNP and macroalbuminuria had been associated with in-hospital mortality in all LVEF groups ( Table 2 ). Coronary artery disease, male gender, and diabetes mellitus predicted in-hospital mortality only in patients with HFmrEF, whereas atrial fibrillation predicted in-hospital mortality only in patients with HFrEF. Older age was an independent predictor of in-hospital mortality in patients with HFrEF and HFpEF.

**Table 2:** – Predictors of in-hospital mortality in HF subtypes

	HFrEF		HFmrEF		HFpEF	
	OR (95% CI)	p-value	OR (95% CI)	p-value	OR (95% CI)	p-value
Coronary artery disease	2.10 (1.55-3.04)	0.065	3.45 (1.23-5.67)	0.043	1.49 (1.14-5.34)	0.089
NT-proBNP	2.68 (1.23-7.75)	<0.001	2.12 (1.34-3.45)	0.011	2.01 (0.09-3.23)	0.022
Age (per 10 years)	1.75 (1.13-3.45)	0.016	1.13 (0.80-1.51)	0.076	3.12 (1.38-4.81)	0.019
Diabetes mellitus	1.21 (0.81-1.43)	0.121	2.34 (1.03-4.16)	0.043	1.20 (0.89-2.55)	0.291
Microalbuminuria	1.94 (0.91-4.21)	<0.001	1.56 (1.19-3.45)	0.001	1.25 (1.12-1.68)	0.124
Macroalbuminuria	2.45 (1.34-5.65)	<0.001	1.92 (1.23-2.98)	0.024	1.66 (1.34-3.84)	0.032
Chronic renal disease	1.15 (1.01-1.33)	0.293	1.32 (1.11-2.77)	0.101	1.23 (0.82-1.56)	0.451
Atrial fibrillation	1.07 (0.83-1.42)	0.013	1.23 (0.89-1.55)	0.234	1.33 (1.18-2.01)	0.098
Male gender	1.22 (0.83-1.88)	0.462	3.31 (1.13-4.23)	0.001	0.89 (0.66-1.39)	0.453

*HFrEF: heart failure with reduced ejection fraction; HFmrEF: heart failure with mid-range ejection fraction; HFpEF: heart failure with preserved ejection fraction; NT-proBNP: N-terminal pro B-type natriuretic peptide.*

### Microalbuminuria and Prognosis

The presence of microalbuminuria on admission has been associated with in-hospital mortality in HFrEF and HFmrEF, but not in HFpEF patients. Patients with microalbuminuria and macroalbuminuria had 1.94-, and 2.45-fold higher risk, respectively, for in-hospital mortality compared to patients with normoalbuminuria in HFrEF.Compared to patients with normoalbuminuria, patients with microalbuminuria and macroalbuminuria had 1.56-, and 1.92-fold higher risk for in-hospital mortality in HFrmEF, respectively.

## Discussion

Our study has several important clinical implications: (i) Of the hospitalized AHF patients, 50% had HFrEF, 11.7% hadHFmrEF, and 38.3% had HFpEF. (ii) 43.4 % of the patients had abnormal UACR at admission to the ED. (iii) The NT-proBNP and UACRvalues and in-hospital mortality rates were the highest in HFrEF patients. (iv) The prevalence of micro- and macroalbuminuria in HFpEF was similar to HFmrEF’s and lower than HFrEF’s. (v) The prevalence of microalbuminuria was 35.2%, 28.8%, and 28.0% in HFrEF, HFpEF, and HFmrEF, respectively. (vi) The microalbuminuria predicted in-hospital mortality in HFmREF and HFrEF, but not in HFpEF.

Cardiovascular and renal diseases share similar comorbidities and risk factors. Extensive cohort studies have shown that increased UACR is associated with the development of HF in the general population.^18 - 20^However, most studies have described the significance of UACR in HFrEF, and studies examining the HF subtypes separately have divergent findings.^21 , 22^In a community-based study, Nayor et al.^21^found that microalbuminuria was associated with an increased risk of incident HFrEF but not HFpEF.^21^In contrast, the PREVEND cohort study showed that higher UACR was more strongly associated with incident HFpEF than HFrEF.^22^In a recent survey of 24433 patients, the association between UACR and HFpEF was greater than HFrEF after 9.3 years of follow-up.^23^

Renal function tests are also associated with adverse outcomes regardless of the severity of the disease in patients with established HF. However, studies investigating the impact of renal dysfunction on prognosis in the different LVEF groups also have conflicting results.^24 , 25^In a meta-analysis, Damman et al.^24^showed that chronic renal dysfunction was a stronger predictor of mortality in HFpEF than in HfrEF.^24^In contrast, in a meta-analysis of twenty-five prospective studies, renal dysfunction was a stronger predictor of mortality in patients with HFrEF than in HFpEF.^25^Both meta-analyses defined chronic renal disease as an eGFR of less than 60 ml/min/1.73m^2^, and studies examining the prognostic value of microalbuminuria or UACR in chronic HF patients with different LVEF groups are much more limited.^26 - 30^In a cross-sectional study, 72 chronic HF were enrolled, and microalbuminuria was observed in 40% of HFpEF and 24% of HFrEF patients (p = 0.04).^26^However, the prognostic impact of microalbuminuria was not evaluated in this study. In the CHARM study, which included chronic HF patients, the prevalence of micro- and macroalbuminuria was 30% and 11%, respectively.^27^When stratifying into different LVEF groups, 31% of the patients with an LVEF ≤40% had microalbuminuria, and 10% had macroalbuminuria. Of the patients with an LVEF >40%, 29% had microalbuminuria, and 12% had macroalbuminuria. The findings of the CHARM study also revealed that albuminuria was a predictor of mortality. The risk associated with UACR was similar in patients with low and preserved LVEF.^27^In the GISSI-HF trial, micro- and macroalbuminuria were observed in 19.9% and 5.4% of the patients, respectively. UACR independently predicted mortality in patients with chronic HF.^28^Nevertheless, as 90.8% of the GISSI-HF patients had an LVEF≤ 40%, a separate analysis for different LVEF groups was not performed. In the CHART-2 study, 2039 chronic HF patients were enrolled.^29^The authors showed that not only microalbuminuria but also subclinical microalbuminuria, which was defined as UACR 10.2–27.3 mg/g, was significantly associated with adverse cardiovascular events as compared with normoalbuminuria, particularly in patients with preserved or mildly reduced eGFR.^28^TOPCAT study included only HFpEF patients to investigate the benefit of spironolactone therapy.^30^In a subgroup analysis of the TOPCAT study, micro- and macroalbuminuria conferred a 1.47- and 1.67-fold increased risk for primary outcomes in HFpEF.^30^

Although the prevalence of renal dysfunction is expected to be higher in AHF patients than patients with chronic HF, few studies assessed albuminuria in the AHF setting. In a prospective study of 115 AHF patients, Koyama et al.^31^showed that 69% of the patients had abnormal UACR at admission (27% had macroalbuminuria, 42% had microalbuminuria).^31^However, on day 7, 10% of the patients had macroalbuminuria, and 30% had microalbuminuria. The resolution of UACR was associated with decreases in NT-pro BNP levels.^31^The frequency of abnormal UACR at admission was 43.4% in our study, lower than the Koyama and colleagues’ study. This difference may be due to younger age and lower comorbidity burden in our study.

Our study demonstrated that the microalbuminuria at admission to ED is an independent predictor of in-hospital mortality in HFmREF and HFrEF, but not in HFpEF.In HFpEF, the prognosis may be more related to comorbidities than in HFmrEF, and HFrEF, where progressive HF with subsequent renal dysfunction may be more pronounced. The relationship between HF and albuminuria is complex. It has a bidirectional nature, and the mechanisms responsible for the relation of microalbuminuria and prognosis in HFrEF and HFmrEF warrant further investigation.

### Study Limitations

Our study is limited by its retrospective design and by having been conducted at a single center. Because daily changes in UACR were not recorded, we could not examine the relationship between alterations in UACR and prognosis. A single spot urine sample was used to determine UACR, which may fluctuate.

## Conclusions

In patients with AHF, microalbuminuria on admission is associated with increased in-hospital mortality in HFmrEF and HFrEF. Further prospective studies are required to explore the role of UACR as a prognostic marker in AHF.
